# Minor Myocardial Scars in Association with Cardiopulmonary Function after COVID-19

**DOI:** 10.1159/000530942

**Published:** 2023-05-12

**Authors:** Tarjei Øvrebotten, Siri Heck, Ingunn Skjørten, Gunnar Einvik, Knut Stavem, Charlotte B. Ingul, Torbjørn Omland, Peder L. Myhre

**Affiliations:** ^a^Division of Medicine, Department of Cardiology, Akershus University Hospital, Lørenskog, Norway; ^b^K.G. Jebsen Center for Cardiac Biomarkers, Institute for Clinical Medicine, University of Oslo, Oslo, Norway; ^c^Department of Diagnostic Imaging, Akershus University Hospital, Lørenskog, Norway; ^d^Department of Respiratory Medicine, Oslo University Hospital, Rikshospitalet, Oslo, Norway; ^e^Pulmonary Department, Akershus University Hospital, Lørenskog, Norway; ^f^Institute for Clinical Medicine, University of Oslo, Oslo, Norway; ^g^Health Services Research Unit, Akershus University Hospital, Lørenskog, Norway; ^h^Department of Circulation and Medical Imaging, Norwegian University of Science and Technology, Trondheim, Norway

**Keywords:** COVID-19, Myocardial scar, Cardiopulmonary performance

## Abstract

**Background:**

Myocardial scars detected by cardiovascular magnetic resonance (CMR) imaging after COVID-19 have caused concerns regarding potential long-term cardiovascular consequences.

**Objective:**

The objective of this study was to investigate cardiopulmonary functioning in patients with versus without COVID-19-related myocardial scars.

**Methods:**

In this prospective cohort study, CMR was performed approximately 6 months after moderate-to-severe COVID-19. Before (∼3 months post-COVID-19) and after (∼12 months post-COVID-19) the CMR, patients underwent extensive cardiopulmonary testing with cardiopulmonary exercise tests, 24-h ECG, and echocardiography. We excluded participants with overt heart failure.

**Results:**

Post-COVID-19 CMR was available in 49 patients with cardiopulmonary tests at 3 and 12 months after the index hospitalization. Nine (18%) patients had small late gadolinium enhancement-detected myocardial scars. Patients with myocardial scars were older (63.2 ± 13.2 vs. 56.2 ± 13.2 years) and more frequently men (89% vs. 55%) compared to those without scars. Cardiorespiratory fitness was similar in patients with and without scars, i.e., peak oxygen uptake: 82.1 ± 11.5% versus 76.3 ± 22.5% of predicted, respectively (*p* = 0.46). The prevalence of ventricular premature contractions and arrhythmias was low and not different by the presence of myocardial scar. Cardiac structure and function assessed by echocardiography were similar between the groups, except for a tendency of greater left ventricular mass in those with scars (75 ± 20 vs. 62 ± 14, *p* = 0.02 and *p* = 0.08 after adjusting for age and sex). There were no significant associations between myocardial scar and longitudinal changes in cardiopulmonary function from 3 to 12 months.

**Conclusion:**

Our findings imply that the presence of minor myocardial scars has limited clinical significance with respect to cardiopulmonary function after COVID-19.

## Introduction

Several studies have reported late gadolinium enhancement (LGE) on cardiovascular magnetic resonance (CMR) imaging after COVID-19 infection. The prevalence of cardiac abnormalities on CMR varies from 3% to 78% after COVID-19 [[1]–[3]], and the majority of studies report scars without a concomitant reduction in left ventricular (LV) function [[4]]. The clinical implication of these minor scars detected after COVID-19 is uncertain. By comparing CMR LGE findings to cardiopulmonary exercise tests (CPETs), echocardiography, 24-h ECG registration, and symptom assessment, we aimed to investigate the clinical implication of myocardial scars following acute COVID-19 infection. We hypothesized that CMR LGE findings after COVID-19 were associated with worse cardiopulmonary function.

## Method

The Patient-Related Outcomes and Lung Function after Hospitalization for COVID-19 (PROLUN), NCT04535154, study [[5]] was a prospective, multicenter observational study, consecutively enrolling unselected adult patients hospitalized with moderate-to-severe COVID-19 between February and June 1, 2020. Patients aged ≥18 years who had been admitted for >8 h with a discharge diagnosis of COVID-19 or viral pneumonia combined with a positive severe acute respiratory syndrome coronavirus 2 polymerase chain reaction test were eligible. A total of 178 patients had cardiopulmonary follow-up at both 3 and 12 months. These patients had extensive cardiopulmonary testing before (∼3 months post-COVID-19, median 98 [IQR 81–126], range 28–187 days) and after (∼12 months post-COVID-19, median 386 [IQR 360–414], range 298–462 days) the CMR. This included CPET, 24-h ECG registration, comprehensive echocardiography, and symptom assessment (online suppl. Fig. 1; for all online suppl. material, see https://doi.org/10.1159/000530942).

The COVID MECH CMR (NCT04314232) study was a prospective, observational study at Akershus University Hospital, enrolling patients with similar inclusion criteria as PROLUN patients, between March 18 and May 4, 2020. Of 128 participants included, 58 patients underwent CMR approximately 6 months, median 166 (IQR 97–203), range 68–237 days, after the index hospitalization [[3]]. Of the 58 patients enrolled in COVID MECH CMR, 50 were also included and participated in the PROLUN follow-up. Since this is a combination of two study cohorts, we did not have a prespecified sample size (online suppl. Fig. 1).

As the purpose of the study was to investigate minor myocardial pathology, minor myocardial scar was defined as myocardial scar without a concomitant reduction in LV ejection fraction below 50%. Severe COVID-19 was defined as admission to the ICU, while admission to the medical ward was considered moderate COVID-19.

CMR was conducted approximately 6 months after the index hospitalization, (median 175 days) between June 24 and November 18, 2020 on a 1.5 MRI scanner (Achieva; Philips Medical Systems, Best, The Netherlands). Details are in the online supplementary. For assessing myocardial scars, we performed two-dimensional phase-sensitive inversion recovery LGE imaging in contiguous 10-mm short-axis slices covering the left ventricle and 3 long-axis views 10 min after injection of 0.15 mmol/kg gadoterate meglumine (Clariscan^®^ Gé, GE Healthcare) [[3]].

The cardiopulmonary exercise testing was performed on a treadmill using Vyntus CPX, Vyaire Medical. Peak oxygen consumption (V̇O_2_ in mL/kg·min), oxygen uptake at anaerobic threshold, oxygen pulse (peak V̇O_2_/heat rate), ventilation at max load (L·min^−1^), and gas exchange (V′E/V′CO2 slope) were recorded and are presented as % of predicted based on age and sex [[6]].

Echocardiography and 24-h ECG registration and dyspnea assessment were performed before and after the CMR, approximately 3 and 12 months after the index hospitalization, according to guideline-recommended methods (online suppl.). We analyzed the following parameters: LV mass, LV end-diastolic volume, LV ejection fraction, LV global longitudinal strain (presented as absolute values), E over e prime, right ventricle free wall longitudinal strain (presented as absolute values), basal right ventricle diameter, tricuspid annular plane systolic excursion, and left atrial volume.

Dyspnea was assessed using the modified Medical Research Council (mMRC) dyspnea scale [[7]], which is a self-rating tool that measures the impact of dyspnea on day-to-day activities on a score from zero (no dyspnea) to 4 (maximum dyspnea). Clinically important dyspnea was defined as mMRC ≥2 (“I walk slower than people of the same age on the level because of breathlessness, or I have to stop for breath when walking at my own pace on the level”).

Baseline characteristics are presented as mean ± standard deviation or medians (1st and 3rd quartile, Q1–Q3) for continuous variables and as absolute numbers and percentages for categorical variables. Change in variables was defined as the first value subtracted from the last value. Clinical data at 3 months and changes in clinical data from 3 to 12 months were compared in patients with and without minor myocardial scars in logistic regression models that were adjusted for age and sex. All statistical analyses were performed using Stata software (version 16, StataCorp., College Station, TX, USA).

## Results

A total of 50 patients with CMR also underwent cardiopulmonary tests at 3 and 12 months after COVID-19 (online suppl. Fig. 1). The patients had a mean age of 58 years with a mean BMI of 28 kg/m^2^; 61% were male, 6% had cardiovascular disease, 17% had hypertension, 4% had chronic obstructive pulmonary disease, 6% had diabetes, and 2% had chronic kidney disease. One patient had major myocardial scar and severe LV dysfunction and was excluded from this analysis, as prespecified. Nine (18%) of the remaining patients had LGE-detected myocardial scars which were all classified as minor, with a mean scar volume 2.2 ± 1.1% (0.7–4.2%). One patient had a combined nonischemic and ischemic (subendocardial) scar, while the rest was nonischemic scars (myocardial and/or epicardial). Patients with myocardial scars were older (63.2 ± 13.2 vs. 56.2 ± 13.2 years) and more frequently men (89% vs. 55%), compared to patients without myocardial scars. Only 3 patients (one with a scar) had a history of cardiovascular disease. Patients with severe COVID-19 were similar in demographics and medical history to patients with moderate disease. There were no significant differences in the prevalence of comorbidities or the severity of COVID-19 hospitalization between the patients with and without myocardial scar (online suppl. Table 1). Participants in the PROLUN study who underwent CMR had overall comparable baseline characteristics to the remaining participants, except fewer non-Caucasians and fewer with hypertension (online suppl. Table 2).

### Myocardial Scar and Echocardiographic Measurements

**Fig. 1:**
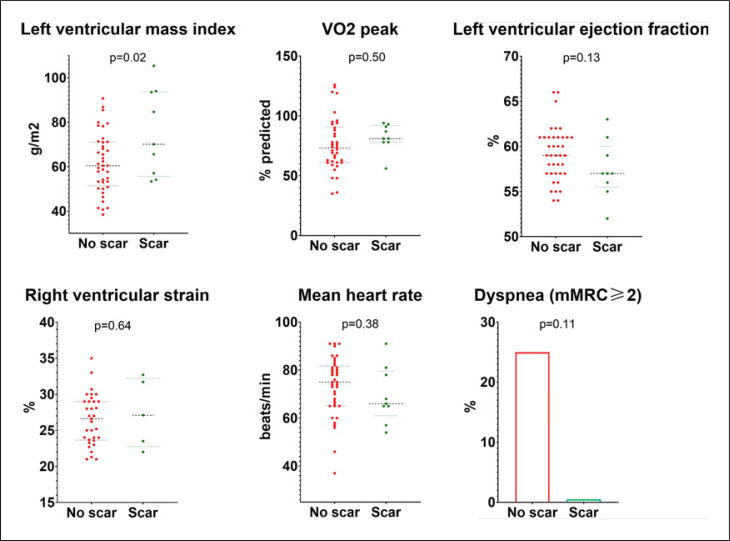
Key measurements by echocardiography, cardiopulmonary exercise testing, 24-h ECG recordings 3 months after COVID-19 in patients with (*n* = 9) and without myocardial scars (*n* = 40) detected by CMR imaging. The thick dashed lines indicate the median value, and the thin dashed lines the quartiles.

Patients with myocardial scars had similar echocardiographic measurements compared to those without scars at 3-month follow-up, except greater LV mass (75.3 ± 19.6 vs. 61.6 ± 13.5 g/m^2^, *p* = 0.02), which was not significant after adjusting for age and sex (*p* = 0.08) (Table 1; Fig. 1). There was no difference in LVEF (59.2 ± 3.0% vs. 57.4 ± 3.2%, *p* = 0.13) or GLS (19.8 ± 1.8% vs. 19.3 ± 2.2%, *p* = 0.52) in patients with and without myocardial scar. When using CMR-derived measures of LV structure and function, the findings were consistent to echocardiographic measurements.

### Myocardial Scar and Cardiopulmonary Performance

The peak oxygen uptake in the overall population was lower than predicted based on age and sex: 77.4 ± 20.8%. There was no difference in peak oxygen update between patients with and without myocardial scars: 82.1 ± 11.5% versus 76.3 ± 22.5% of predicted (*p* = 0.46). There was also no difference in peak oxygen pulse between patients with and without myocardial scars: 92.7 ± 18.7% versus 93.6 ± 14.1% of predicted (*p* = 0.89), respectively, or V′_E_/V′_CO2_ slope (*p* = 0.13), ventilation at maxload (*p* = 0.92), or peak oxygen uptake at anaerobic threshold (*p* = 0.66) (Table 1). Scar size was not associated with peak oxygen uptake (B 0.002 [95% CI −0.05–0.01], *p* = 0.50) or other measures of cardiopulmonary performance.

### Myocardial Scar and Electrocardiographic Measurements

Only 2 patients had non-sustained ventricular arrhythmias, and both were among patients with myocardial scars: 5% versus 0% (*p* = 0.49). Patients with myocardial scars had a greater burden of premature ventricular contractions (PVC; median [IQR] per 24 h): 37 (17–154) versus 3 (1–34) (*p* = 0.04). The mean heart rate was similar between the groups: 73 versus 69/min, *p* = 0.38. In total, 4 patients had Q-waves, 1 with and 3 without myocardial scars. None had bundle branch block on ECG.

### Dyspnea and Clinical Features

There was no significant difference in self-reported dyspnea (mMRC ≥2) at 3 months by the presence of myocardial scars: 0% with scar versus 25% without scar (*p* = 0.11). We found no difference between the two groups when comparing clinical features at the index hospitalization for COVID-19, including oxygen saturation, respiratory rate, heart rate, blood pressure, and cardiac biomarkers (Table 1).

### Changes in Cardiopulmonary Function from 3 to 12 Months

There were no between-group differences in changes in cardiopulmonary function, echocardiographic measurements, or dyspnea from 3 months to 12 months after COVID-19 in patients with and without myocardial scars (Table 2).

## Discussion

This study found no difference in cardiovascular function, cardiopulmonary performance, or reported dyspnea 3 months after COVID-19 between patients with and without minor myocardial scars. Furthermore, there was no association between myocardial scar and change in cardiopulmonary function between 3 and 12 months.

The prevalence of myocardial scars in the general population (pre-COVID-19) is estimated to be 7.8% (mean age: 69 years) [[8]], and even higher proportions have been reported in athletes [[9]]. In our study, 18% of patients had myocardial scars after hospitalization for COVID-19, which is lower than in other studies [[1], [4]]. Differences in inclusion criteria (i.e., elevated cardiac troponin [[4]]), and the timing of CMR [[1]], may explain some of these differences. The patients in our cohort have previously been demonstrated to have comparable prevalence (18% vs. 15%) and size (2.2 ± 1.1% vs. 1.4 ± 1.3%) of myocardial scars to healthy individuals from the general population [[8]]. The majority of patients with scars were male, which coincides with previous research [[8]]. The risk of myocardial scar may increase after COVID-19 due to diffuse myocardial inflammation from SARS-CoV-2. In more severe cases, COVID-19 can cause acute myocarditis, but this only occurs in 2–4 per 1,000 patients hospitalized for COVID-19 [[10]]. Most myocardial scars detected after COVID-19 are small and without a concomitant reduction in LV function [[4]], and it is unknown whether SARS-CoV2 causes more myocardial scars than other respiratory viruses. We have previously demonstrated that myocardial scars detected after COVID-19 are not associated with the severity of the infection [[3]]. We now extend these findings by demonstrating limited associations between minor myocardial scars and cardiopulmonary function and dyspnea after COVID-19. These findings suggest that the clinical implication of detecting such scars is limited, except for a tendency of a greater burden of PVC and nonsustained ventricular tachycardia which should be investigated in future studies. The extent of myocardial scars is associated with poor outcomes in different cardiomyopathies [[11]], while the presence of myocardial scars in the general population seems to be less prognostically important [[12]]. Although we did not assess the long-term prognosis associated with myocardial scars after COVID-19, we demonstrate no significant association with established prognostic markers such as peak oxygen uptake. There was no association between scar size and cardiopulmonary performance in our study. However, only 9 of the included patients had myocardial scar, and we, therefore, had limited power to detect a potential association.

This study examines the association between the presence of scar and several cardiopulmonary outcomes and thus is at risk of false-positives due. However, we did not identify any clinically relevant associations and did therefore not adjust for multiple testing.

The cardiac status before COVID-19 is unknown, and whether cardiac scarring is caused by COVID-19 is therefore uncertain. A recent study with serial CMR before and after COVID-19 infection showed that all the detected scars were present before the COVID-19 infection [[13]]. Moreover, our cohort had a similar prevalence of myocardial scars as studies from the general population [[8]]. The modest sample size and the fact that all patients were hospitalized and enrolled during the first wave of COVID-19 before vaccination and the evolution of different strains of COVID-19 limit the extrapolation of our results to contemporary care and to non-hospitalized patients.

## Conclusion

With technological advances and the increasing availability of CMR, more patients will have an incidental discovery of minor myocardial scars. Whether or not such findings alone require further workup is controversial. Despite a modest sample size, our study addresses an important issue where there is limited research. Our findings may inspire future research and introduce caution in interpreting findings of minor myocardial scars since minor myocardial scars after COVID-19 seem to have little clinical significance and is primarily related to older age and male sex.

## Statement of Ethics

COVID MECH, COVID CMR, and PROLUN were approved by the Regional Ethics Committee for South‐Eastern Norway (#20/02873; #20/05884; #125384). Consent was given by returning a written signed consent form. Data were stored and post-processed in Services for Sensitive Data (TSD; University of Oslo), in compliance with the Norwegian Personal Data Act and Health Research Act.

## Conflict of Interest Statement

P.L.M. has served on advisory boards or received speaker fees from Amgen, AstraZeneca, Bayer, Boehringer Ingelheim, Novartis, and Novo Nordisk, unrelated to this work. C.I. has received fees for lectures from Bayer A.G. T.O. has received consultancy and speaker honoraria from Abbott Diagnostics, Roche Diagnostics, and Novartis and research support via Akershus University Hospital from Thermo Fisher BRAHMS, HyTest Ltd., Biomedica, Abbott Diagnostics, Novartis, Singulex, SomaLogic, and Roche Diagnostics. T.O. also has financial interests in CardiNor A.S., which holds the license to commercialize secretoneurin. All the other authors have no conflicts of interest to declare.

## Funding Sources

This study received funding from Akershus University Hospital and LHL Hospital Gardermoen (The National Association for Heart, Lung diseases and the Norwegian Health Association).

## Author Contributions

T.Ø. performed most of the echocardiographic studies at the 12-month follow-up, interpreted all echocardiographic images, and performed the statistical analysis with P.L.M. S.H. analyzed all CMR images. I.S. performed and analyzed the CPET. G.E. and K.S. were responsible for running the PROLUN study. C.B.I. was responsible for the cardiac sub-study in PROLUN and performed echocardiographic studies at the 3-month follow-up. T.O. was the PI of COVID CMR. P.L.M. was a major contributor in writing the manuscript and running the statistical analysis. All the authors read and approved the final manuscript.

## Data Availability Statement

The datasets generated and/or analyzed during the current study are not publicly available due to privacy or ethical restrictions but are available from the corresponding author on reasonable request.

## Supplementary Material

Supplementary data

## Figures and Tables

**Table 1:** Cardiac structure and function, cardiopulmonary performance, and symptoms 3 months after discharge for COVID-19 in patients with and without minor myocardial scar

	No scar, *n* = 40	Scar, *n* = 9	*p* value	*p* adj. age	*p* adj. age and sex
Echocardiography					
LV mass indexed, g/m^2^	61.6±13.5	75.3±19.6	0.02	0.04	0.08
LV end diastolic volume indexed, mL/m^2^	50±10.4	48.6±14.0	0.67	0.33	0.30
LVEF, %	59.2±3.0	57.4±3.2	0.13	0.06	0.07
LV GLS, %	19.8±1.8	19.3±2.2	0.52	0.33	0.42
E/e'	8.4±2.7	8.9±3 0.1	0. 67	0.84	0.92
RVLS, %	26.5±3.5	27.3±4.6	0.64	0.80	0.79
RVD, cm	3.6±0.5	3.6±0.6	0.98	0.37	0.45
TAPSE, cm	2.4±0.4	2.5±0.7	0.76	0.71	0.56
LA volume indexed, mL/m^2^	23.9±5.4	25.5±9.5	0.54	0.74	0.80
Dyspnea					
mMRC ≥2	8 (25%)	0 (0.0%)	0.11	0.07	0.04
CPET					
VO_2_ peak, % predicted	76.3±22.5	82.1±11.5	0.46	0.63	0.69
O_2_-pulse peak, % predicted	92.7±18.7	93.6±14.1	0.89	0.79	0.91
V_E_/V_CO2_ slope	29.2±4.8	31.3±4.6	0.13	0.27	0.10
V_E_ at max. load, L·min^−1^	79.1±35.2	80.3±20.7	0.92	0.56	0.57
VO_2_ at, % predicted of VO_2_ max	48.8±12.3	46.9±7.0	0.66	0.44	0.86
ECG					
24-h mean heart rate, /min	73.4±12.3	69.4±11.8	0.38	0.52	0.85
24-h NSVT	2 (5.0%)	0	0.49	0.48	0.52
24-h median (Q1, Q3) PVC	3 [1, 34]	37 [17, 154]	0.041	0.35	0.59
Q-waves	3 (7.5%)	1 (11.1%)	0.73	0.86	0.98
Clinical features at index hospitalization					
Heart rate, /min	88±16	80±15	0.24	0.29	0.30
Respiratory rate, /min	29±12	25±8	0.43	0.55	0.50
Oxygen saturation, %	94±3	94±2	0.89	0.61	0.65
Oxygen supply	7 (17.5%)	3 (33.3%)	0.29	0.29	0.10
Systolic blood pressure, mm Hg	130±18	136±15	0.43	0.74	0.75
TnT max, ng/L	10 [6–16]	13 [12–16]	0.13	0.32	0.65
NT-proBNP max, ng/L	126 [35–312]	142 [101–449]	0.28	0.67	0.58
CRP max, mg/dL	125 [55–260]	120 [60–180]	0.90	0.94	0.97

Reported as mean ± SD, median (Q1, Q3), or number (%). *p* values are calculated from logistic regression analyses. LVMi, left ventricular mass index; LVEF, left ventricular ejection fraction; LV GLS, left ventricular global longitudinal strain; RVLS, right ventricle free wall longitudinal strain; RVD, basal right ventricle diameter; TAPSE, tricuspid annular plane systolic excursion; LAVi, left atrial volume index; VO_2_ peak, maximal oxygen consumption; O2-pulse peak, maximal oxygen pulse; VE, expired ventilation; VCO2, carbon dioxide output; AT, anaerobic threshold; NSVT, nonsustained ventricular tachycardia; PVC, premature ventricular contractions; VO_2_, oxygen uptake; VE, expired ventilation; O2, oxygen; VCO2, carbon dioxide output; AT, anaerobic threshold; SD, standard deviation.

**Table 2:** Changes in cardiopulmonary performance and symptoms from 3 to 12 months after hospitalization for COVID-19

	No myocardial scar, *n* = 40	Myocardial scar, *n* = 9	*p* value	*p* adj. age	*p* adj. age, sex
Echocardiography					
Δ LV mass indexed, g/m^2^	0.0±7.4	−1.5±4.2	0.58	0.78	0.86
Δ LV end diastolic volume indexed, mL/m^2^	−1.1±6.0	1.8±10.3	0.30	0.27	0.42
Δ LVEF, %	−0.2±2.8	−0.4±5.2	0.85	0.65	0.62
Δ LV GLS	0.2±1.5	−0.6±3.2	0.46	0.63	0.42
Δ E/e'	−0.4±2.6	−0.4±1.4	0.96	0.99	0.92
Δ RV GLS, %	−0.7±2.6	−0.5±6.2	0.94	0.89	0.78
Δ RVD, cm	−0.0±0.4	−0.1±0.2	0.35	0.46	0.73
Δ TAPSE, cm	0.02±0.2	0.10±0.3	0.30	0.39	0.25
Δ LAVi, mL/m^2^	−0.7±3.6	−2.9±5.0	0.19	0.27	0.21
Cardiopulmonary exercise test					
ΔVO_2_ peak (% predicted)	4.4±11.4	1.2±6.3	0.41	0.33	0.27
ΔO_2_-pulse peak (% predicted)	5.8±11.6	0.1±14.2	0.21	0.18	0.22
ΔV′_E_/V′_CO2_ slope	0.3±4.5	−0.3±2.7	0.68	0.52	0.36
ΔV′_E_ at max. load, L·min^−1^	0.4±17.2	7.2±16.2	0.23	0.27	0.34
ΔV′_O2_ at AT, % predicted of VO_2_ max	2.7±8.8	4.7±5.9	0.51	0.59	0.44

Reported as mean ± SD. *p* values were calculated from logistic regression analyses. LVMi, left ventricular mass index; LVEF, left ventricular ejection fraction; LV GLS, left ventricular global longitudinal strain; RVLS, right ventricle free wall longitudinal strain; RVD, basal right ventricle diameter; TAPSE, tricuspid annular plane systolic excursion; LAVi, left atrial volume index; VO_2_ peak, maximal oxygen consumption; O_2_-pulse peak, maximal oxygen pulse; V_E_, expired ventilation; V_CO2_, carbon dioxide output; AT, anaerobic threshold; NSVT, nonsustained ventricular tachycardia; PVC, premature ventricular contractions; VO_2_, oxygen uptake; V_E_, expired ventilation; O_2_, oxygen; V_CO2_, carbon dioxide output; AT, anaerobic threshold; SD, standard deviation.
