# Supplementary material for: Minor Myocardial Scars in Association with Cardiopulmonary Function after COVID-19
Source: Cardiology. 2023 May 12;148(4):300–6. doi: 10.1159/000530942 (PMC10614250; doi:10.1159/000530942)
Supplement: Supplementary file 1 — Supplementary data [file crd-0148-0300-s01.docx]

Supplemental Material

to

**Minor myocardial scars in association with cardiopulmonary function after COVID-19**

**SUPPLEMENTAL METHODS**

*Cardiovascular magnetic resonance imaging*

Left ventricular (LV) volume and ejection fraction and mass was measured using 8 mm short-axis slices, acquired through steady-state-free precession sequences with dedicated software (cvi42, v5.11.4, Circle Cardiovascular Inc., Calgary, Canada) in accordance with the Society for Cardiovascular Magnetic Resonance guidelines^1^. LV volumes included the papillary muscles and trabeculations. LGE was defined as signal intensity >5 standard deviations from normal myocardium. Signal intensity thresholding was performed semiautomatically. Myocardial scars were classified as ischemic or non-ischemic according to the SCMR recommendations. The scar was classified as ischemic when LGE involved the subendocardium and was consistent with a coronary artery perfusion territory. LGE patterns limited to the mid-wall or epicardium and sparing the subendocardium were classified as non-ischemic^1^**.** Further details can be found in the article by Myhre et al^2^

*Echocardiography*

LV ejection fraction was derived using Simpson`s biplane method. Diastolic LV end diastolic volume and left atrial volume were calculated using apical two and four-chamber views and indexed against body surface area. Systolic (S`) and diastolic (e`) pulsed tissue Doppler velocities were obtained from the lateral wall of the tricuspid annulus, and the septal and lateral wall of the mitral annulus. Mitral inflow velocities (E-wave, A-wave, E/A-ratio) were obtained with pulsed wave Doppler.

Tricuspid annular plane systolic excursion was measured in an apical four-chamber view. Maximal tricuspid regurgitation velocity and inferior vena cava dimension were used to calculate the estimated systolic pulmonary artery pressure .

Images used in the evaluation of LV global longitudinal strain were obtained from apical two, three- and four-chamber views, and right ventricular free wall strain images were obtained from a focused four-chamber view^3^. All speckle tracking quantifications were performed using semi-automatic software^4^. All images were stored and analyzed offline with the use of GE EchoPAC PC SWO version 204. The same investigator analyzed all images.

### *Electrocardiogram (ECG)*

All ECG`s were evaluated for pathological Q-waves and bundle branch block.

### *24-hour electrocardiogram*

A 24-hour electrocardiogram (ECG) was recorded using machines from Schiller Medilog FD12 Plus (Germany) and Philips DigiTrak XT (Germany). All recordings were reviewed by a cardiologist.

**Suppl.Table 1** Baseline characteristics of the study population 3 months after hospitalization for COVID-19, stratified by the presence of minor myocardial scar on cardiac magnetic resonance imaging.

|  | NO MYOCARDIAL SCAR | MYOCARDIAL SCAR |  |
| --- | --- | --- | --- |
|  | **n=40** | **n=9** | **p-value** |
|  |  |  |  |
| Age, years | 56.2 ± 13.2 | 63.2±13.2 | 0.16 |
| Male sex | 22 (55%) | 8 (88.9%) | 0.06 |
| White race | 27 (67.5%) | 7 (77.8%) | 0.74 |
| Body mass index, kg/m^2^ | 27.9±4.4 | 27.3±3.9 | 0.33 |
| Obesity (BMI > 30) | 11 (28%) | 3 (33%) | 0.94 |
| Diabetes Mellitus | 4 (10%) | 0 (0.0%) | 0.32 |
| Hypertension | 6 (15%) | 2 (22.2%) | 0.62 |
| Cardiovascular disease | 2 (5%) | 1 (11%) | 0.62 |
| Chronic pulmonary disease | 2 (5%) | 0 | 0.49 |
| Chronic kidney disease | 2 (5%) | 0 | 0.48 |
| Previous smoking | 17 (42.5%) | 5 (55.6%) | 0.48 |
| Index hospitalization for COVID-19 | | | |
| Length of stay (days) | 7 [3,12 ] | 6 [5 , 9 ] | 0.87 |
| ICU admission | 11 (27.5%) | 2 (22.2%) | 0.75 |
| Ventilator | 8(20%) | 1(11.1%) | 0.53 |

**Suppl. Table 2** Baseline characteristics of patients included in the PROLUN study that were not included compared to those who were included in the current cardiovascular magnetic imaging study

|  | Not included | Included |  |
| --- | --- | --- | --- |
|  | **n=127** | **n=49** | **p-value** |
|  |  |  |  |
| Age, years | 58.5±13.6 | 57.5±13.4 | 0.68 |
| Male sex | 74 (58%) | 30 (60%) | 0.72 |
| White race | 109 (86%) | 34 (69%) | 0.01 |
| Body mass index, kg/m^2^ | 28.3±4.7 | 27.8±4.3 | 0.50 |
| Obesity | 39 (31%) | 14 (29%) | 0.78 |
| Diabetes Mellitus | 11 (9%) | 4 (8.3%) | 0.57 |
| Hypertension | 48 (39%) | 8 (17%) | 0.005 |
| Cardiovascular disease | 15 (12%) | 3 (6%) | 0.26 |
| Chronic pulmonary disease | 3 (2%) | 2 (4.1%) | 0.54 |
| Chronic kidney disease | 1 (6.3%) | 2 (4.3%) | 0.75 |
| Previous smoking | 62 (49%) | 22 (45%) | 0.64 |
| *Index hospitalization for COVID-19* | | | |
| Length of stay (days) | 6 [3 , 11 ] | 6 [3 , 11 ] | 0.79 |
| ICU admission | 22 (17%) | 9 (18%) | 0.22 |
| Ventilator | 14(11%) | 9(18%) | 0.22 |

**Suppl Figure 1**


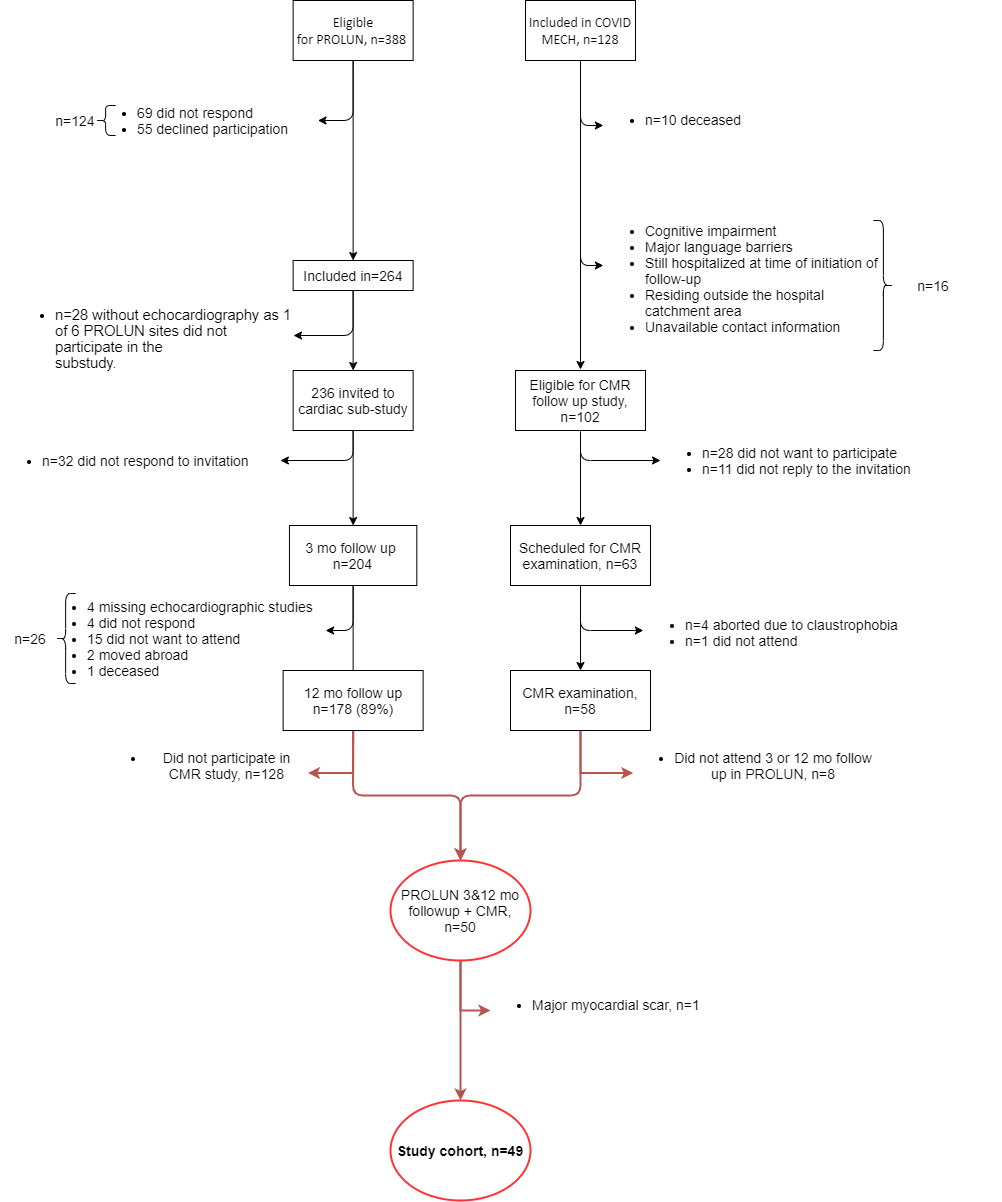


**SUPPLEMENTARY REFERENCES**

1. Schulz-Menger J, Bluemke DA, Bremerich J, et al. Standardized image interpretation and post-processing in cardiovascular magnetic resonance - 2020 update. *Journal of Cardiovascular Magnetic Resonance*. 2020;22(1)doi:10.1186/s12968-020-00610-6

2. Myhre PL, Heck SL, Skranes JB, et al. Cardiac pathology 6 months after hospitalization for COVID-19 and association with the acute disease severity. *The American heart journal*. 2021;242:61-70. doi:10.1016/j.ahj.2021.08.001

3. Mitchell C, Rahko PS, Blauwet LA, et al. Guidelines for Performing a Comprehensive Transthoracic Echocardiographic Examination in Adults: Recommendations from the American Society of Echocardiography. *J Am Soc Echocardiogr*. Jan 2019;32(1):1-64. doi:10.1016/j.echo.2018.06.004

4. Recommendations for Cardiac Chamber Quantification by Echocardiography in Adults: An Update from the American Society of Echocardiography and the European Association of, Cardiovascular Imaging. *Eur Heart J Cardiovasc Imaging*. 2016;17(4):412-412. doi:10.1093/ehjci/jew041
